# Placenta percreta avec envahissement vésical

**DOI:** 10.11604/pamj.2014.18.88.4631

**Published:** 2014-05-26

**Authors:** Younes Essatara, Hicham Benazzouz

**Affiliations:** 1 Clinique Urologique, CHU Ibn Sina, Rabat, Maroc

**Keywords:** Placenta percreta, vessie, myomètre, Placenta percreta, bladder, myometrium

## Image en medicine

Le placenta percreta se définit par une invasion placentaire étendue avec atteinte conjuguée du myomètre et de la séreuse utérine et par un envahissement plus ou moins important des organes pelviens adjacents. Les complications maternelles sont représentées par les risques hémorragiques, de rupture utérine, d'envahissement vésical, de sténose ou fistule urétérale et digestive. Une femme de 32 ans avec comme ATCD quatre grossesses dont 2 accouchements par césarienne a été hospitalisée à 33 SA devant l'apparition de métrorragie minime, une échographie/doppler obstétricale et une IRM avaient montré un placenta recouvrant ayant un aspect perccreta. La patiente a développé par la suite à 5 jours de son admission des métrorragies foudroyantes de grande abondance ayant nécessité son admission d'urgence au bloc opératoire. L'exploration chirurgicale avait identifié un placenta totalement recouvrant antero-postérieur envahissant le segment inférieur et le trigone /rétro trigone vésical, une hystéroraphie a permis l'extraction d'un nouveau-né de sexe féminin, puis réalisation d'une hystérectomie sub-total hautement hémorragique laissant en place un placenta inextirpable hautement adhérant a la face postérieur de la vessie et constatation d'un délabrement vésical important. Après repérage des deux méats urétéraux, intubation et montée de sondes urétérales des deux cotés extériorisée en trans-vésico-pariétal puis fermeture laborieuse sur sonde double courant de la vessie dont la paroi postérieur ne se résumait qu'à une fine paroi muqueuse reposant sur le placenta, la patiente a présenté pendant l'intervention plusieurs épisodes d'hypotension et a été transfusée en per et post opératoire de 8 culots globulaires.

**Figure 1:**
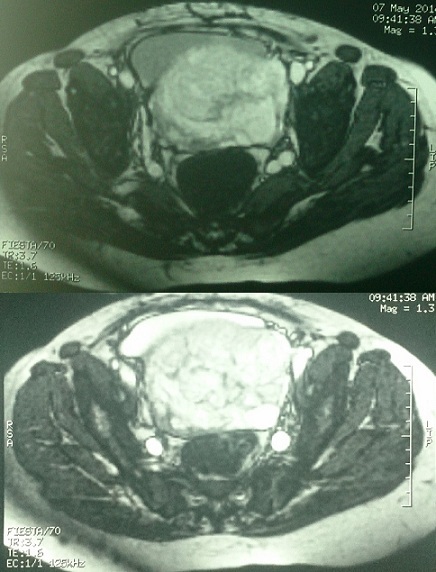
Placenta percreta avec envahissement vésical

